# *Azospirillum* spp. from Plant Growth-Promoting Bacteria to Their Use in Bioremediation

**DOI:** 10.3390/microorganisms10051057

**Published:** 2022-05-20

**Authors:** María Antonia Cruz-Hernández, Alberto Mendoza-Herrera, Virgilio Bocanegra-García, Gildardo Rivera

**Affiliations:** 1 Laboratorio Interacción Ambiente Microorganismo, Centro de Biotecnología Genómica, Instituto Politécnico Nacional, Reynosa 88710, Mexico; macruzh@ipn.mx (M.A.C.-H.); amendoza@ipn.mx (A.M.-H.); vbocanegg@hotmail.com (V.B.-G.); 2 Laboratorio de Biotecnología Farmacéutica, Centro de Biotecnología Genómica, Instituto Politécnico Nacional, Reynosa 88710, Mexico

**Keywords:** *Azospirillum*, xenobiotic, bioremediation

## Abstract

Xenobiotic contamination, a worldwide environmental concern, poses risks for humans, animals, microbe health, and agriculture. Hydrocarbons and heavy metals top the list of toxins that represent a risk to nature. This review deals with the study of *Azospirillum* sp., widely reported as plant growth-promoting bacteria in various cultures. However, its adaptation properties in adverse environments make it a good candidate for studying remediation processes in environments polluted with hydrocarbons and heavy metals. This review includes studies that address its properties as a plant growth promoter, its genomics, and that evaluate its potential use in the remediation of hydrocarbons and heavy metals.

## 1. Introduction

The enormous increase in the world population requires increased agricultural productivity and improved food quality to cover basic needs [1]; however, this has caused the indiscriminate application of chemical fertilizers, leading to pollution. Heavy metal toxicity has also become an important agronomic problem, mainly because of intense anthropogenic activities [2]. Among the sources of contamination caused by these xenobiotics, we can mention hydrocarbons produced by drilling, extraction, conduction, and transformation activities, conditions that have caused soil and water contamination mainly due to spills, leaks, seepage, and sludge [3]. In the case of heavy metals, reports mainly mention the use of agricultural pesticides that contain heavy metals [4], industrial discharges, mining [5], dust and soil in urban areas [6], transportation, damming, and wastewater disposal and runoff [7,8]. These problems generate harmful effects that cause environmental and human health degradation [9]. However, microbial remediation research refers to intense changes in microbe diversity induced by environmental pollution and adaptation mechanisms that allow microorganisms to adapt to environments with xenobiotic contaminants [10,11]. Therefore, this review summarizes some microbial remediation studies, mainly the removal of hydrocarbons, and studies in which bacteria have been isolated, including *Azospirillum* sp., which can tolerate heavy metals.

## 2. Plant Growth Promotion

Plant growth promotion is a characteristic of plant growth-promoting rhizobacteria (PGPR) [12]. These bacteria positively influence the growth and development of plants [13]. The direct growth stimulation mechanisms of these bacteria are based on facilitating the absorption of nutrients and synthesizing or regulating plant hormones [14,15]. Indirect mechanisms of PGPR influence plant growth and comprise a wide range of mechanisms that prevent or suppress plant diseases [1,16].

**Figure 1:**
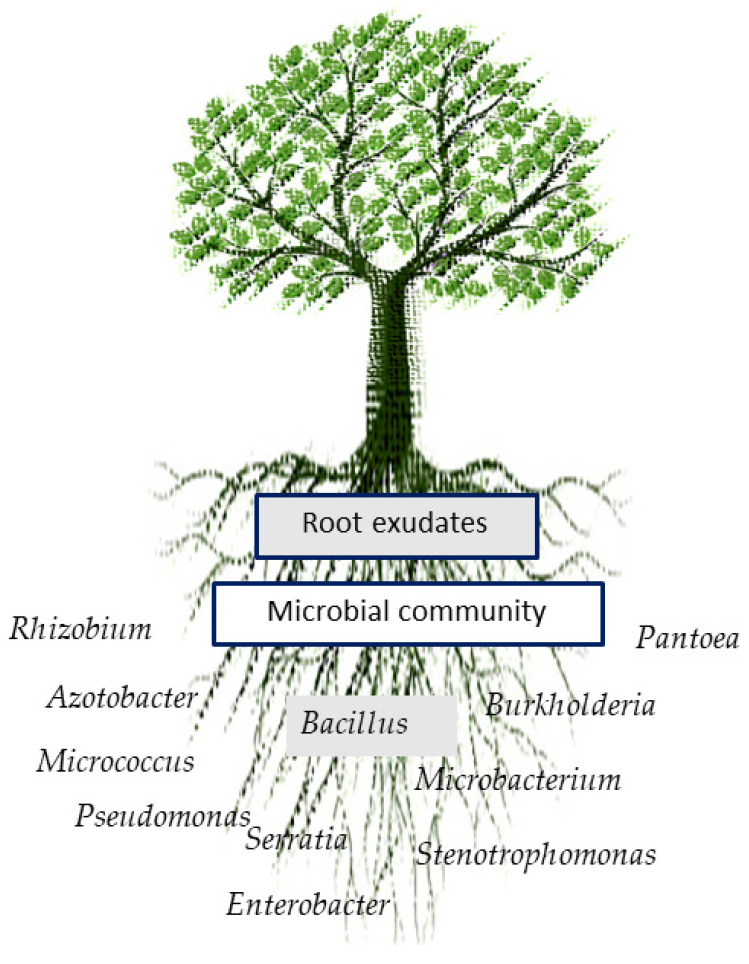
Common microbial community in the rhizosphere.

Numerous microorganisms such as *Rhizobium* [17], *Azotobacter* [18], *Burkholderia* [19], *Enterobacter* [20], *Pantoea* [21], *Bacillus* [22], *Pseudomonas* [23], *Stenotrophomonas* [24], *Micrococcus*, *Microbacterium* [25], and *Serratia* [26] have been proven excellent agricultural growth-stimulating agents (Figure 1).

Some microorganisms have been used as bioinoculants or microbial inoculants to increase crop productivity without causing contamination [27,28]. This use has been considered one of the contributions of biotechnology and modern microbiology because it is a viable option to help reverse the effects of contamination [29]. Among these bacteria, we can mention species of the genus *Azospirillum*. These bacteria secrete phytohormones such as auxins, cytokinins, and gibberellins that produce changes in plant root architecture, inducing the development of adventitious roots [30,31] and root hairs on their host plants which is beneficial due to root growth stimulation [11]. PGPR inoculation has been widely used in agriculture [32], and the genus *Azospirillum* is one of the most studied [27,30,33,34].

## 3. *Azospirillum*

Bacteria belonging to the genus *Azospirillum* are free-living microbes that promote plant growth (PGPB). They affect the growth and yield of numerous plant species, many of agronomic and ecological importance [11]. The most accepted theory regarding the mechanism of action of *Azospirillum* is its growth promotion, which includes nitrogen fixation [35,36] and phytohormone, polyamine, and trehalose production [30]. The mode of action of *Azospirillum* is multiple, and the importance of each of these mechanisms can vary depending on soil and climate conditions and the solubilization of minerals such as iron and phosphorus, which the plant uses [30]. These mechanisms eventually produce larger, and in many cases, more productive plants [33,34]. *Azospirillum* has improved crop yields of wheat, corn, rice, and sugar cane [30]. It has also been used in chili pepper, fruit trees, and cacti [30]. In the case of the genus *Azospirillum*, 25 species isolated from different niches have been reported (Table 1). Most of these species have been isolated from roots of wild plants [37,38] and cultured [39,40,41] from aquatic environments [42,43,44] and contaminated areas [44,45,46,47,48]. 

The first two species described and the most studied are *Azospirillum lipoferum* (*A. lipoferum*) and *Azospirillum brasilense* (*A. brasilense*) [30,31,62].

## 4. Genetics of *Azospirillum* Species

With the advent of molecular biology, some of these species have been sequenced, complementing the knowledge of the range of genes of this bacterium that involves its different characteristics, such as those of plant growth promotion. Species of the genus *Azospirillum* vary relative to their genome size; in the case of *A. irakense*, it is 4800 kb, and in *A. lipoferum*, 9600 kb. *A. brasilense* about 7000 kb. Megaplasmids are characteristic in their genome; some are linear [63,64]. The presence of these megaplasmids is common. They are one of the first genomic characteristics reported for the genus *Azospirillum*. Their number varies between species, with 7 to 8 replicons and even 10 commonly found [63]. Each strain shows a unique profile composed of one to six plasmids with sizes that range from 100 kb to 1.7 Mb, which is present in *Azospirillum* strains in one copy per cell. In addition to plasmids in *Azospirillum* strains, minichromosomes have also been reported. Interestingly, in *A. brasilense*, the genome comprises multiple chromosomes with replicons of 600, 1000, and 1700 kb. The existence of multiple chromosomes in *A. brasilense* was corroborated, but a 2500 kb extra chromosome was reported [65,66].

## 5. Degradation of Xenobiotics by *Azospirillum* Species

**Figure 2:**
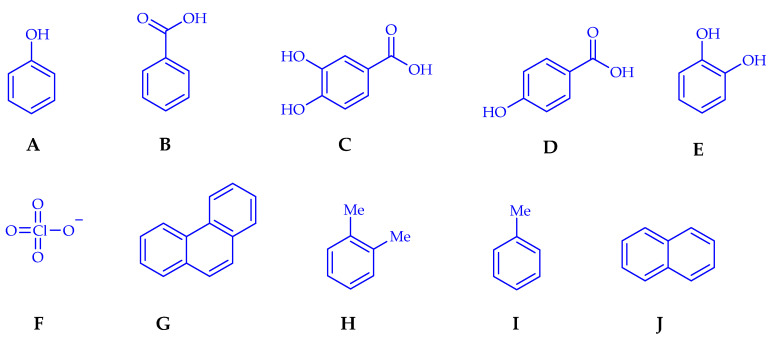
Chemical structures degraded by *Azospirillum* strains (**A**) phenol, (**B**) benzoate, (**C**) protocatechuate, (**D**) 4-hydroxybenzoate, (**E**) catechol, (**F**) perchlorate, (**G**) phenanthrene, (**H**) xylene, (**I**) toluene and (**J**) naphthalene.

Bacteria of this genus possess versatile carbon and nitrogen metabolic pathways adapted to competitive environments where desiccation and nutrient limitations predominate [67]. Although rhizobacteria have been widely used as plant growth promoters, there are reports on using *Azospirillum* strains in pollutant degradation processes [10]. One example is the work by Barkovskii et al., which evaluated the degradation of phenol (Figure 2A) and benzoate (Figure 2B) using 31 strains of *A. brasilense* and *A. lipoferum* isolated from rhizospheres and rhizoplanes of different plants. Their results showed that nine strains degraded benzoate and three strains, phenol [68].

On the other hand, López de Victoria and Lowell reported a study that evaluated the chemotaxis of *A. lipoferum* 59b, *A. brasilense* Sp 7, and *A. brasilense* Cd to aromatic compounds such as benzoate (A), protocatechuate (B), 4-hydroxybenzoate (C), and catechol (D) (Figure 2). They reported that *A. brasilense* Sp 7 responded to much lower levels of aromatic compounds than *A. lipoferum*. Additionally, *A. brasilense* Cd was more sensitive to all the assayed aromatic compounds than *A. brasilense* Sp 7, clearly observing a degree of specificity between the evaluated *Azospirillum* strains [69].

Eckford et al. isolated five nitrogen-fixing bacteria from fuel-contaminated Antarctic soil; among these, *A. brasilense*. Their results show the importance of the association between the isolated diazotroph bacteria, such as *Azospirillum*, and bacteria hydrocarbon degraders that are not diazotrophs because they provide nitrogen in Antarctic soil, a condition that is beneficial in nitrogen-poor soil [70].

Nozawa et al. evaluated the composition of the bacterial community in microcosms enriched with perchlorate (Figure 2F) and acetate or hydrogen. After this, they carried out a partial sequencing of 16S rRNA genes recovered from the microcosms. Phylogenetic analysis indicated the presence of *Azospirillum* spp. Therefore, their results emphasize perchlorate bioremediation by native microbial communities in soil [71].

Likewise, Muratova et al. evaluated the tolerance of 33 *Azospirillum* strains from different species, including *A. brasilense,* in a culture medium with 1% light crude oil as a carbon source. They found that *A. lipoferum* SR42 and *A. brasilense* SR80 degraded crude oil 57.5% and 56.5% after 14 days of incubation. Afterward, *A. brasilense* SR80 strain was used to evaluate its associative ability. Their results showed that the strain was chemotactically attracted to wheat exudates, colonized the roots, and produced indole acetic acid; likewise, the synthesis of indole acetic acid was not inhibited by oil [72].

A study of phytoremediation in Mexico by Miranda-Martínez et al. evaluated five bacterial strains isolated from oil-contaminated soils that degrade phenanthrene (Figure 2G) and are atmospheric nitrogen fixers. The study also included *A. brasilense* Cd and *A. halopraeferens* isolated from non-contaminated soils. Using sand inoculated with phenanthrene with and without the German grass, *Echinochloa polystachya* (HBK) Hitch, they determined their population dynamics. They evaluated three cases: (a) inoculation with a bacteria consortium composed of strains isolated from contaminated soils and *A. halopraeferens,* (b) inoculation with *A. brasilense* Cd, and (c) without bacterial inoculation. The authors mention that 60 days after inoculation, phenanthrene degradation was greater (*p* < 0.05) in plants inoculated with the bacteria consortium (59.01%) and *A. brasilense* Cd (57.02%) compared to the control without plants (41.7%) [73]. This study again demonstrates the importance of evaluating numerous strains because the response varies between species and even between strains.

Commonly, *Azospirillum* strains isolated from the rhizosphere of crops were used in xenobiotic tolerance and degradation assays; however, in 2008, the first *Azospirillum* strain was isolated from hydrocarbon-contaminated soil. This was the case in Young et al., who isolated a strain from oil-contaminated soil characterized by a polyphasic taxonomic approach. A comparative analysis of the 16S rRNA gene sequence showed that the isolate was phylogenetically related to the genus *Azospirillum*. It was proposed as a new strain named *A. rugosum* [47].

On the other hand, Lin et al. isolated a strain in discarded tar. After characterization, it was reported as a new strain named *A. picis* [48]. Likewise, Zhou et al. isolated a nitrogen-fixing strain, SgZ-5^T^, from a microbial fuel cell (MFC) characterized by a polyphasic approach. They stated that it represented a novel species, *A. humicireducens* [53].

Another study performed by Cruz-Hernández et al. [74] used nineteen *A. brasilense* strains to evaluate their tolerance in vitro to xenobiotics, such as phenanthrene (A), xylene (B), toluene (C), and naphthalene (D) (Figure 2). They characterized biosurfactant production and searched for genes related to aromatic compound degradation using the RAST program. Their results showed that the strains produced biosurfactants; however, when carrying out tolerance tests to xylene, toluene, phenanthrene, and naphthalene, no growth of the evaluated strains was observed. However, nineteen coding sequences related to the degradation of aromatic compounds were recorded; eleven are associated with the metabolism of central aromatic intermediates and five with peripheral catabolic pathways whose function is associated with quinate, benzoate, salicylate, gentisate, and toluene degradation pathways. This study was the first of isolated *A. brasilense* strains in Northeast Tamaulipas, Mexico [74].

Biosurfactants are compounds that are mostly produced by fungi and bacteria. The release of biosurfactants is one strategy used by microorganisms to influence hydrocarbon absorption and hydrophobic compounds in general [75,76]. The structure of these compounds allows the solubilization of hydrocarbons to make them degradable [77,78]). These molecules have emulsifying and dispersing properties. When the carbon source is partially soluble or insoluble in water, these molecules are synthesized with tensoactive properties that favor the biodegradation of insoluble substrates [79].

A recent study by Wu et al. reported two isolates, RWY-5-1-1^T^ and ROY-1-1-2, obtained from an oil production mixture of Yumen Oilfield in Gansu, China. These isolates were phenotypically, genotypically, and chemotaxonomically characterized to determine their taxonomic position. The isolates were reported as a novel species, *A. oleiclastium*. The authors assessed their diazotrophy and detected genes related to hydrocarbon degradation and biosurfactant production. Therefore, hydrocarbon degradation tests and biosurfactant production analyses were performed. The results showed that the oil degradation rate of strain RWY-5-1-1^T^ after 14 days of incubation in NFB broth was 36.2%. In the emulsification experiment, stable emulsions were formed using diesel oil, kerosene, and soybean oil. These results indicate that biosurfactants could exist in the fermentation broth. This study demonstrated that the new strains of *Azospirillum* can degrade oil associated with plant growth promotion [61].

Recently, Maimona et al. established a bioremediation system of plant microbiomes to treat crude-oil contamination. They isolated ten strains of PGPR from oil-contaminated soil in Pakistan. Based on the plant growth-promoting characteristics and surfactant production, they selected two strains, *Pseudoarthrobacter phenanthrenivorans* (MS2) and *A. oryzae* (MS6). They inoculated both strains and a combination into rhizospheric soil of maize in crude oil-contaminated soil to establish the plant-microbiome system. The hydrocarbon degradation efficiency of this system was 38.5%. An analysis of degradation products by GC-MS revealed the presence of low molecular weight hydrocarbons in the treated soil compared to untreated soil. Together with nitrogen-fixing activity, the new isolates can promote certain ecological interactions between plants and microbes in strong hydrocarbon contamination conditions. This finding emphasizes the study of *Azospirillum* strains to degrade hydrocarbons because reports are scarce [80].

## 6. Tolerance of Heavy Metals in *Azospirillum* Species

Heavy metal contamination has become a serious environmental problem because they distribute widely in ecosystems [81]. Plant Growth Promoting bacteria have been widely used to improve plant performance and improve the stress caused by xenobiotic contamination [13]. One of these is the rhizobacterium, *A. brasilense*, in which studies of its plant growth-promoting activities have been performed. One study of these bacteria, reported by Langenbach et al., evaluated the effects of silver (Ag), cadmium (Cd), zinc (Zn), and lead (Pb) on the growth and nitrogenase activity of *Azospirillum* spp. They found that at metal concentrations of 0.1 and 1 ppm, growth and nitrogenase activity, respectively, were suppressed. Nitrogen fixation by *Azospirillum,* as well as the production of growth regulators, are desirable characteristics in this genus. Pb (1 ppm) inhibited 25% of acetylene reduction activity (ARA), and growth was not inhibited by lead oxide or chloride. Zn (1 ppm) inhibited 50% of ARA, remaining constant with concentration increases.

In contrast, Ag and Cd inhibited 50% ARA at lower concentrations (0.4 ppm) than those needed to inhibit growth by 50% (2.4 and 6 ppm, respectively). Resistance was observed after 24 h of incubation with Cd [82]. Bacteria of the *Azospirillum* genus are nitrogen fixers and producers of growth-regulating substances, so they benefit the plants with which they interact.

Belimov and Dietz evaluated the effect of cadmium chloride (CdCl_2_ at 50 μM) in barley seedlings inoculated with *A. lipoferum* 137, *Arthrobacter mysorens* 7, *Agrobacterium radiobacter* 10, and *Flavobacterium* sp. The assays were performed in hydroponics using quartz sand as a substrate. In the plants that grew in the absence of Cd, bacteria increased the content of nutrients, such as phosphorus (P), magnesium (Mg), calcium (Ca), iron (Fe), manganese (Mn), and sodio (Na) in the roots and/or shoots. In plants treated with Cd, inoculation of the bacteria caused a positive effect on root length and biomass. The positive changes in the composition of elements caused by the bacteria were less marked in plants treated with Cd. The total amount of nutrients taken up by the inoculated plants significantly increased. The Cd content in the inoculated plants did not vary, except (for an increase) in the roots with the addition of *A. lipoferum* 137. Thus, the results showed that the bacteria were able to partially reduce the toxicity of Cd in the barley plants by improving the absorption of nutritional elements [83].

After that, Belimov et al. reported a study evaluating the rhizobacteria *A. lipoferum* 137, *Arthrobacter mysorens* 7, *Agrobacterium radiobacter* 10, and *Flavobacterium* sp., which showed resistance to heavy metals, Pb and Cd (except strain L30, which was found sensitive to Cd). In pot and field experiments, seed inoculation with bacteria improved barley plant growth and nutrient uptake from Pb- and Cd-contaminated soil. Inoculation also prevented Pb and Cd accumulation in barley plants, thus mitigating the toxic effect of these heavy metals on plants [84].

Akond and Khan isolated *Azospirillum* strains from rice root samples; three were identified as *A. brasilense* (N2, N18, and N21), and two as *A. amazonense* (N5 and N15), in which their growth, nitrogen fixation, and their effect on growth in rice plants supplemented with Cd, chromium (Cr), Pb, mercury (Hg), Ag, and Zn were analyzed. The results showed that the nitrogen-fixing potential in all the strains gradually decreased with the increase in heavy metal concentrations. Additionally, each strain had a gradual decrease in growth with the increase in the concentration of each heavy metal. The strains showed tolerance at a concentration of 0.1 and 0.2 ppm of Hg. The N18 strain showed tolerance at a concentration of 0.5 ppm of Ag; however, it was sensitive to Zn at 100 ppm (minimum concentration used). The strains N5, N15, and N21 were tolerant to Zn at a concentration of 500 ppm. Finally, this study found a difference in nitrogen fixation in the *Azospirillum* strains since fixation decreased with an increased concentration of the evaluated heavy metals [85].

Similarly, Kamnev et al. evaluated two *A. brasilense* strains Sp245 (endophyte) and Sp7 (no endophyte), against cobalt (Co), copper (Cu), and Zn at different concentrations (up to 0.2 mmol/L). They used Fourier transform infrared (FTIR) spectroscopy to control the compositional characteristics of whole cells. Their results showed that heavy metals induce a greater accumulation of the polyester compound, poly-3-hydroxybutyrate (PHB), in the Sp7 strain. Sp245 has a response less pronounced. However, both strains showed a lower production of indole-3-acetic acid (auxin). The authors suggested that this behavior responds to different environmental conditions from each strain [86].

On the other hand, Lyubum et al., evaluated the effect produced by inoculation of the bacteria *A. brasilense* Sp245 in wheat plants of the variety ‘Saratovskaya 29,’ using a medium contaminated with arsenic (As). The assays were performed in hydroponics, evaluating three concentrations, 75, 750, and 7500 μg/L^−1^. The evaluated parameters were root length, bud size, and total dry matter. The results showed that the As concentration did not affect the development of root length in the plants inoculated with the bacteria. Likewise, the plants inoculated with *A. brasilense* decreased root length; however, a greater formation of side roots was observed due to Indole acetic acid (IAA) production. This finding is an important characteristic of species of the genus *Azospirillum* that allows the plants to have better root development offering major access to nutrients in the soil [11]. The As concentrations that were evaluated in the medium influenced plant weight. The plants showed a minimum weight at an As concentration of 7500 μg/L^−1^. Regarding the effect caused by *A. brasilense* inoculation, no significant difference was observed in plant weight; however, As (III) absorption decreased in the plants inoculated with *Azospirillum* compared to those not inoculated, reducing the initial As (III) concentration used by 75% [87].

Vezza et al. evaluated the viability of *A. brasilense* Cd strain in As contaminated agricultural soils to evaluate its response to arsenate (As V) and arsenite (As III). The results showed that this bacterium tolerated As concentrations frequently found in soils. Additionally, their characteristics, such as colonization, growth promotion and N2 fixation, are not altered. Therefore, this strain could be an option in the bioremediation of soils [88].

On the other hand, Ogar et al. evaluated the development of alfalfa (*M. sativa*) and hawkweed (*H. pilosela*) using an unsterilized substrate containing Zn-Pb and a sterilized substrate to eliminate the microorganisms present. The treatments were established under greenhouse conditions. The first treatment included plants inoculated with arbuscular mycorrhizal fungi (AMF) and nitrogen-fixing bacteria. In the second treatment, nitrogen-fixing bacteria were inoculated in both types of substrates (sterilized and unsterilized). The results showed differences in plant growth according to the substrate used, with less growth in the sterilized substrate than in the unsterilized substrate. However, in the treatments using the sterile substrate inoculated with AMF and *Azospirillum* sp, significant differences were found in the parameters of biomass and growth of *H. pilosela* compared to the experiments where only diazotroph bacteria (*Azospirillum* and *Nostock edaphicum*) were inoculated [89].

Ganesh et al. evaluated the toxic effects of Cr on the growth and yield of rice plants. Toxicity was mitigated with microbial inoculants, especially *Azospirillum*. The field experiment was performed with the rice variety ASD 16 in Cr-contaminated soil inoculated with microorganisms, including *Azospirillum*. The highest rice morphological and yield parameters were recorded in the contaminated soil mixed with the *Azospirillum* application [90].

Rojas et al. evaluated the growth of two strains, *A. brasilense* and *Glomus intraradices,* using compost with heavy metals as As, Cd, Cr, Cu, nickel (Ni), and Pb. The results showed that heavy metals did not have a negative effect on the growth of both bacteria. *A. brasilense* was tolerant to As concentrations of 0.4375 mg kg^−1^, Pb (62.375 mg kg^−1^), and Cu (91.1875 mg kg^−1^). However, it was susceptible to Cd at 0.5 mg kg^−1^ [91].

On the other hand, Arora et al. evaluated the bioremediation potential of AMF and *Azospirillum* in *Panicum virgatum* (switchgrass) against Pd and Cd. The authors compared the growth parameters and bioremediation potential of AMF (*Glomus mossei, G. fasciculatum*, and *Gigaspora margarita*) and *Azospirillum* at different Pb and Cd concentrations. The results showed that the fungi and *Azospirillum* increased the root length, branches, surface area, and biomass of roots and buds. A significant difference in the bioaccumulation coefficient (BAC) was also seen. Greater absorption of heavy metals was found in the soil of plants inoculated with *Azospirillum*, followed by AMF and *Azospirillum* + AMF. Therefore, grass inoculated with AMF + *Azospirillum* can be an effective phytoremediator (phytoaccumulator/phytostabilizer), although heavy metals are concentrated mainly in the underground biomass [92].

A study reported by Balakrishnan et al. evaluated the concentrations of heavy metals (Cd, Cr, Cu, Ni, Pb, Fe, Mn, and Zn) in the soil and rhizosphere of *Avicennia marina* in mangroves from India and tested for resistant bacteria. Rhizosphere soil showed higher concentrations of metals (Cd, Fe, Mn, and Zn from 6.0 to 16.7% Cr, Cu, Ni, and Pb from 1.7 to 2.8%). The results showed that the site with the highest contamination of heavy metals presented the highest number of isolated bacteria, including strains of *Pseudomonas, Azotobacter, Shewanella*, and *A. brasilense*, which showed tolerance to Cr and Cu. The authors showed that bacteria resistant to heavy metals could be used to indicate heavy metal contamination and the bioremediation of contaminated sites [93].

Xu et al. evaluated the effect of the bacteria *Bacillus subtilis* and *A. brasilense* (Indole butyric acid producers) on Cd accumulation in native *Arabidopsis thaliana* plants compared to a mutant plant not sensitive to abscisic acid (ABA). Their results found that native plants inoculated with *Azospirillum* and *Bacillus* increased ABA levels, resulting in better development. In the case of the mutant plant inoculated with the bacteria, a smaller effect relative to Cd reduction was observed. The authors mention that the ABA produced and provided by the bacteria could help suppress Cd absorption in the roots of plants due to the inhibition of IRT1, an Fe and Cd transporter. This work emphasizes the importance of hormones produced by these bacteria and their role in strategies that reduce contaminants in crops, in this case, Cd [94].

On the other hand, Armendariz et al., evaluated the effect of Ar (As V o As III) at a concentration of 25 mM in soy plants (*Glycine max* cv. DM 4670) inoculated with the diazotrophic bacteria *Bradyrhizobium japonicum* E109 and *A. brasilense* Az39. Initially, they evaluated the viability of the two strains in vitro in the presence of Ar at a concentration of 25 μM of As V and As III. Afterward, they performed an in vivo assay with soy plants under controlled conditions at a concentration of 25 μM of As (As V and As III), evaluating the total nitrogen content with the Kjeldahl method. The results showed that in in vitro assays, the mortality of *B. japonicum* E109 and *A. brasilense* Az39 decreased 21% in As V and 13–27% in As III. The authors suggest that the phytohormone IAA produced by *Azospirillum* could be a cause, indicating a possible synergetic effect between the evaluated bacteria. This finding could be because reports mention that *B. japonicum* can use IAA as a carbon source. In the in vivo assays with As, it was found that the plants inoculated with both bacteria had greater growth, a greater number of nodules, and a reduction in the translocation of As to the aerial parts. The nitrogen content of uninoculated plants did not change, while plants with As inoculated with *A. brasilense* Az39 showed a greater nitrogen content, although it did not significantly differ. In plants inoculated with *Bradyrhizobium japonicum* E109, As caused a decrease in nodules, affecting nitrogen fixation since it was low (around 20–25%) compared to plants inoculated with *A. brasilense* Az39 [95].

Pan et al. evaluated the development of *Brassica chinensis* L. grown in Cd-contaminated soil inoculated with the abscisic acid (ABA)-generating bacteria, *A. brasilense,* and *Bacillus subtilis*. The results showed a biomass increase of 40%-79% and 43%-77% lower Cd concentrations than control plants that were not inoculated with bacteria. The inoculation of *A. brasilense* and *Bacillus subtilis* improved the levels of antioxidant-related compounds and nutritional quality [96].

Recently, Vázquez et al., performed an experiment to evaluate the effect of inoculating wheat plants containing Cd with *A. brasilense* Az39 under controlled conditions. The results showed that in bacteria inoculated plants, the effect of Cd on plant growth was mitigated. Despite having a more developed root system, inoculated plants had lower Cd levels than uninoculated plants. The authors mention that this could be due to the production of bacterial siderophores, which causes a lower availability of Cd and the formation of a siderophore-Cd complex [97].

## 7. Conclusions

The effects of xenobiotics on plants inhibit growth and decrease physiological and biochemical activities and plant function. The effects of the persistence and bioavailability of hydrocarbons and/or heavy metals depend on several factors, such as environmental conditions, pH, and the affected plant species. However, studies have reported the resistance mechanisms of microorganisms on the toxic effects of pollutants. These mechanisms can act in synergy with the plants, achieving effective phytoremediation. Microbial activity at the contaminated site acts as an indicator of plant growth and bioremediation. Aerobic or anaerobic bacteria used in bioremediation can use hydrocarbons as a carbon and energy source for growth and reproduction. In addition, enzymes such as dioxygenases, peroxidases, and catalases are produced through their metabolism. They oxidize hydrocarbons and transform or degrade them to less-toxic compounds. *Azospirillum* strains have been widely studied and are used as inoculants in various crops due to their plant growth-promoting properties. However, their beneficial effects depend on their viability and functionality in adverse environmental conditions, such as growth in environments contaminated by xenobiotics. Interestingly, different studies show that *Azospirillum* strains can degrade xenobiotics such as hydrocarbons and show tolerance to different heavy metals, highlighting the importance of continuing studies aimed at their use individually or through the formation of bacterial consortia.

## Figures and Tables

**Table 1:** *Azospirillum* species reported from different sources and countries.

Name	Country	Source	Reference
*Azospirillum largimobile* (*A. largimogile*)	Senegal	Grass	[37]
*Azospirillum orizae* (*A. orizae*)	Japan	Rice	[38]
*Azospirillum lipoferum* (*A. lipoferum*)	Brasil	Wheat	[39]
*Azospirillum irakense* (*A. irakense*)	Iraq	Rice	[40]
*Azospirillum formosense* (*A. formosense*)	Taiwan	Rice	[41]
*Azospirillum thiophilum* (*A.tiophilum*)	Russia	Water	[42]
*Azospirillum griseum* (*A. griseum*)	China	Agua	[43]
*Azospirillum oleicastium* (*A. oleicastium*)	China	Oil	[44]
*Azospirillum rugosum* (*A. rugosum*)	Taiwan	Contaminated soil	[45]
*Azospirillum picis* (*A. picis*)	Taiwan	Tar	[46]
*Azospirillum fermentarium* (*A. fermentarium*)	Taiwan	Fermenter	[47]
*Azospirillum humicireducens* (*A. humicireducens*)	China	Microbial fuel cell	[48]
*Azospirillum brasilense* (*A. brasilense*)	Brazil	Grass	[49]
*Azospirillum halopraeferens* (*A. halopraeferens*)	Pakistan	Grass	[50]
*Azospirillum doebereinerae* (*A. doebereinerae*)	Germany	Grass	[51]
*Azospirillum melinis* (*A. melinis*)	China	Grass	[52]
*Azospirillum canadense* (*A. canadense*)	Canada	Corn	[53]
*Azospirillum zeae* (*A. zeae*)	Canada	Corn	[54]
*Azospirillum palatum* (*A. palatum*)	China	Soil	[55]
*Azospirillum soli* (*A. soli*)	Taiwan	Agricultural soil	[56]
*Azospirillum agricola* (*A. agricola*)	Taiwan	Agricultural soil	[57]
*Azospirillum palustre* (*A. palustre*)	Russia	Soil	[58]
*Azospirillum ramasamyi* (*A. ramasamyi*)	Korea	Fermented bovine products	[59]
*Azospirillum baldaniorum* (*A. baldoniorum*)	Brazil	Rhizosphere	[60]
*Azospirillum thermophilum* (*A. thermophilum*)	China	Hot spring	[61]

## Data Availability

Not applicable.

## References

[1] Asad S.A., Farooq M., Afzal A., West H. (2019). Integrated phytobial heavy metal remediation strategies for a sustainable clean environment—A review. Chemosphere.

[2] Fan X.H., Zhang S.A., Mo X.D., Li Y.C., Fu Y.Q., Liu Z.G. (2017). Effects of plant growth-promoting rhizobacteria and N source on plant growth and N and P uptake by tomato grown on calcareous soils. Pedosphere.

[3] Núñez R.R., Lorenzo M., Ortiz E., Oramas J. (2010). Biorremediación de la contaminación de petróleo en el mar. Rev. Electron. Agencia Medio Ambiente.

[4] Wuana R.A., Okieimen F.E. (2011). Heavy metals in contaminated soils: A review of sources, chemistry, risks and best available strategies for remediation. ISRN Ecol..

[5] Chun S.J., Kim Y.J., Cui Y., Nam K.H. (2021). Ecological network analysis reveals distinctive microbial modules associated with heavy metal contamination of abandoned mine soils in Korea. Environ. Pollut..

[6] Long Z., Zhu H., Bing H., Tian X., Wang Z., Wang X., Wu Y. (2021). Contamination, sources and health risk of heavy metals in soil and dust from different functional areas in an industrial city of Panzhihua City, Southwest China. J. Hazard. Mater..

[7] Sun Z., Mou X., Tong C., Wang C., Xie Z., Song H., Sun W., Lv Y. (2015). Spatial variations and bioaccumulation of heavy metals in intertidal zone of the Yellow River estuary, China. Catena.

[8] Xu F., Qiu L., Cao Y., Huang J., Liu Z., Tian X., Li A., Yin X. (2016). Trace metals in the surface sediments of the intertidal Jiaozhou Bay, China: Sources and contamination assessment. Mar. Pollut. Bull..

[9] Gerhardt K.E., MacNeill G.J., Gerwing P.D., Greenberg B.M., Ansari A., Gill S., Guy R., Lanza G., Newman L. (2017). Phytoremediation of Salt-Impacted Soils and Use of Plant Growth-Promoting Rhizobacteria (PGPR) to Enhance Phytoremediation. Phytoremediation.

[10] Pandey S., Ghoshb S., Misra A.K. (2009). Synthesis of a Trisaccharide and a Tetrasaccharide from the Cell-Wall Lipopolysaccharides of *Azospirillum brasilense* S17. Synthesis.

[11] Pii Y., Mimmo T., Tomasi N., Terzano R., Cesco S., Crecchio C. (2015). Microbial interactions in the rhizosphere: Beneficial influences of plant growth-promoting rhizobacteria on nutrient acquisition process. A review. Biol. Fert. Soils.

[12] Umesha S., Singh P.K., Singh R.P., Singh R.L., Monda S. (2018). Microbial Biotechnology and Sustainable Agriculture. Biotechnology for Sustainable Agriculture.

[13] Lu Q., Weng Y., You Y., Xu Q., Li H., Li Y., Liu H., Du S. (2020). Inoculation with abscisic acid (ABA)-catabolizing bacteria can improve phytoextraction of heavy metal in contaminated soil. Environ. Poll..

[14] Kong Z., Glick B.R. (2017). The role of plant growth-promoting bacteria in metal phytoremediation. Adv. Microb. Physiol..

[15] Backer R., Rokem J.S., Ilangumaran G., Lamont J., Praslickova D., Ricci E., Subramanian S., Smith D.L. (2018). Plant growth-promoting rhizobacteria: Context, mechanisms of action, and roadmap to commercialization of biostimulants for sustainable agriculture. Front. Plant Sci..

[16] Goswami D., Thakker J.N., Dhandhukia P.C. (2016). Portraying mechanics of plant growth promoting rhizobacteria (PGPR): A review. Cogent Food Agric..

[17] Olanrewaju O.S., Glick B.R., Babalola O.O. (2017). Mechanisms of action of plant growthpromoting bacteria. World J. Microbiol. Biotechnol..

[18] Kannapiran E., Ramkumar V.S. (2011). Inoculation effect of nitrogen-fixing and phosphate-solubilizing bacteria to promote growth of black gram (*Phaseolus mungo* Roxb; Eng). Ann. Biol. Res..

[19] Mishra P.K., Joshi P., Suyal P., Bisht J.K., Bhatt J.C. (2014). Potential of Phosphate Solubilising Microorganisms in Crop Production. Bioresources for Sustainable Plant Nutrient Management.

[20] Mamta R.P., Pathania V., Gulati A., Singh B., Bhanwra R.K., Tewari R. (2010). Stimulatory effect of phosphate-solubilizing bacteria on plant growth, stevioside and rebaudioside-A contents of Stevia rebaudiana Bertoni. Appl. Soil Ecol..

[21] Sulbarán M., Pérez E., Ball M.M., Bahsas A., Yarzábal L.A. (2009). Characterization of the mineral phosphate-solubilizing activity of Pantoea aglomerans MMB051 isolated from an iron-rich soil in southeastern Venezuela (Bolívar State). Curr. Microbiol..

[22] Santos S., Neto I.F.F., Machado M.D., Soares H.M.V.M., Soares E.V. (2014). Siderophore production by *Bacillus megaterium*: Effect of growth phase and cultural conditions. Appl. Biochem. Biotechnol..

[23] Mehnaz S., Arora N.K. (2013). Secondary Metabolites of *Pseudomonas aurantiaca* and Their Role in Plant Growth Promotion. Plant Microbe Symbiosis: Fundamentals and Advances.

[24] Jiang Z., Chen M., Yu X., Xie Z. (2016). 7-Hydroxytropolone produced and utilized as an iron-scavenger by *Pseudomonas donghuensis*. BioMetals.

[25] Cruz-Hernández M.A., Reyes-Peralta J., Mendoza-Herrera A., Rivera G., Bocanegra-García V. (2021). Characterization of a *Microbacterium* sp. Strain isolated from soils contaminated with hydrocarbons in the Burgos basin, Mexico. Rev. Int. Contam. Ambient..

[26] Lalitha S., Dhanarajan A. (2017). Plant Growth–Promoting Microbes: A Boon for Sustainable Agriculture. Sustainable Agriculture towards Food Security.

[27] Kaushal M., Wani S.P. (2016). Plant-growth-promoting rhizobacteria: Drought stress alleviators to ameliorate crop production in drylands. Ann. Microbiol..

[28] Vasseur-Coronado M., Dupré du Boulois H., Pertot I., Puopolo G. (2021). Selection of plant growth promoting rhizobacteria sharing suitable features to be commercially developed as biostimulant products. Microbiol. Res..

[29] Abbasian F., Lockington R., Mallavarapu M., Naidu R.A. (2015). Comprehensive Review of Aliphatic Hydrocarbon Biodegradation by Bacteria. Appl. Biochem. Biotechnol..

[30] Bashan Y., Bashan L.E., Sparks D.L. (2010). How the Plant Growth-Promoting Bacterium *Azospirillum* Promotes Plant Growth—A Critical Assessment. Advances in Agronomy.

[31] Souza R., Ambrosini A., Passaglia L.M. (2015). Plant growth-promoting bacteria as inoculants in agricultural soils. Genet. Mol. Biol..

[32] Dos Santos J., Maranho L. (2018). Rhizospheric microorganisms as a solution for the recovery of soils contaminated by petroleum: A review. J. Environ. Manag..

[33] Díaz-Zorita M., Fernandez-Canigia M.V., Bravo O.A., Berger A., Satorre E.H., Cassan F.D., Okon Y., Creus C.M. (2015). Field Evaluation of Extensive Crops Inoculated with *Azospirillum* sp.. Handbook for Azospirillum, Technical Issues and Protocols.

[34] García J.E., Maroniche G., Creus C., Suárez-Rodríguez R., Ramírez-Trujillo J.A., Groppa M.D. (2017). In vitro PGPR properties and osmotic tolerance of different *Azospirillum* native strains and their effects on growth of maize under drought stress. Microbiol. Res..

[35] Machado H.B., Funayama S., Rigo L.U., Pedrosa F.O. (1991). Excretion of ammonium by *Azospirillum brasilense* mutants resistant to ethylenediamine. Can. J. Microbial..

[36] Santos K.F.D.N., Moure V.R., Hauer V., Santos A.R.S., Donatti L., Galvao C.W., Pedrosa F.O., Souza E.M., Wassem R., Steffens M.B.R. (2017). Wheat colonization by an *Azospirillum brasilense* ammonium-excreting strain reveals upregulation of nitrogenase and superior plant growth promotion. Plant Soil.

[37] Ben Dekhil S., Cahill M., Stackebrandt E., Sly L.I. (1997). Transfer of Conglomeromonas largomobilis subsp. largomobilis to the genus *Azospirillum* as *Azospirillum largomobile* comb. nov., and elevation of *Conglomeromonas largomobilis* subsp. parooensis to the new type species of *Conglomeromonas*, *Conglomeromonas parooensis* sp. nov. Syst. Appl. Microbiol..

[38] Xie C.-H., Yokota A. (2005). *Azospirillum oryzae* sp. nov., a nitrogen-fixing bacterium isolated from the roots of the rice plant Oryza sativa. Int. J. Syst. Evol. Microbiol..

[39] Beijerinck M.W. (1925). Uber ein Spirillum, welches frein Stickstoff binden kann?. Zentralbl. Bakteriol. Parasitenkd. Infektionskr..

[40] Khammas K.M., Ageron E., Grimont P.A.D., Kaiser P. (1991). *Azospirillum irakense* sp. nov., a nitrogen-fixing bacterium associated with rice roots and. Res. Microbiol..

[41] Lin S.Y., Shen F.T., Young L.S., Zhu Z.L., Chen W.M., Young C.C. (2012). *Azospirillum formosense* sp. nov., a diazotroph from agricultural soil. Int. J. Syst. Evol. Microbiol..

[42] Lavrinenko K., Chernousova E., Gridneva E., Dubinina G., Akimov V., Kuever J., Lysenko A., Grabovich M. (2010). *Azospirillum thiophilum* sp. nov., a diazotrophic bacterium isolated from a sulfide spring. Int. J. Syst. Evol. Microbiol..

[43] Yang Y., Zhang R., Feng J., Wang C., Chen J. (2019). *Azospirillum griseum* sp. nov., isolated from lakewater. Int. J. Syst. Evol. Microbiol..

[44] Wu D., Zhang X.J., Liu H.C., Zhou Y.G., Wu X.L., Nie Y., Kang Y.Q., Cai M. (2021). *Azospirillum oleiclasticum* sp. nov, a nitrogen-fixing and heavy oil degrading bacterium isolated from an oil production mixture of Yumen Oilfield. Syst. Appl. Microbiol..

[45] Young C.C., Hupfer H., Siering C., Ho M.J., Arun A.B., Lai W.A., Rekha P.D., Shen F.T., Hung M.H., Chen W.M. (2008). *Azospirillum rugosum* sp. nov., isolated from oil-contaminated soil. Int. J. Syst. Evol. Microbiol..

[46] Lin S.Y., Young C.C., Hupfer H., Siering C., Arun A.B., Chen W.M., Lai W.A., Shen F.T., Rekha P.D., Yassin A.F. (2009). *Azospirillum picis* sp. nov., isolated from discarded tar. Int. J. Syst. Evol. Microbiol..

[47] Lin S.Y., Liu Y.C., Hameed A., Hsu Y.H., Lai W.A., Shen F.T., Young C.C. (2013). *Azospirillum fermentarium* sp. nov., a nitrogen-fixing species isolated from a fermenter. Int. J. Syst. Evol. Microbiol..

[48] Zhou S., Han L., Wang Y., Yang G., Li Z., Hu P. (2013). *Azospirillum humicireducens* sp. nov., a nitrogen-fixing bacterium isolated from a microbial fuel cell. Int. J. Syst. Evol. Microbiol..

[49] Tarrand J.J., Krieg N.R., Dobereiner J. (1979). A taxonomic study of the *Spirillum lipoferum* group, with descriptions of a new genus, *Azosporillum* gen. nov. and two species, *Azospirillum lipoferum* (Reijerinckia) comb., nov. and *Azospirillumbrasilense* sp. nov. Can. J. Microbiol..

[50] Reinhold B., Hurek T., Fendrik I., Pot B., Gillis M., Kersters K., Thielemans S., De Ley J. (1987). *Azospirillum halopraeferens* sp. nov., a nitrogen-fixing organism associated with roots of kallar grass (*Leptochloa fusca* (L.) kunth). Int. J. Syst. Bacteriol..

[51] Eckert B., Weber O.B., Kirchhof G., Halbritter A., Stoffels M., Hartmann A., Kirchhof G., Halbritter A., Eckert B. (2001). *Azospirillum doebereinerae* sp. nov., a nitrogen-fixing bacterium associated with the C4-grass Miscanthus. Int. J. Syst. Evol. Microbiol..

[52] Peng G., Wang H., Zhang G., Hou W., Liu Y., Wang E.T., Tan Z. (2006). *Azospirillum melinis* sp. nov., a group of diazotrophs isolated from tropical molasses grass. Int. J. Syst. Evol. Microbiol..

[53] Mehnaz S., Weselowski B., Lazarovits G. (2007). *Azospirillum canadense* sp. nov., a nitrogen-fixing bacterium isolated from corn rhizosphere. Int. J. Syst. Evol. Microbiol..

[54] Mehnaz S., Weselowski B., Lazarovits G. (2007). *Azospirillum zeae* sp. nov., a diazotrophic bacterium isolated from rhizosphere soil of Zea mays. Int. J. Syst. Evol. Microbiol..

[55] Zhou Y., Wei W., Wang X., Xu L., Lai R. (2009). *Azospirillum palatum* sp. nov. isolated from forest soil in Zhejiang province, China. J. Gen. Appl. Microbiol..

[56] Lin S.Y., Hameed A., Liu Y.C., Hsu Y.H., Lai W.A., Shen F.T., Young C.C. (2015). *Azospirillum soli* sp. nov., a nitrogen-fixing species isolated from agricultural soil. Int. J. Syst. Evol. Microbiol..

[57] Young C.C., Lin S.Y., Hameed A., Liu Y.C., Hsu Y.H., Huang H.I., Lai W.A. (2016). *Azospirillum agricola* sp. nov., a nitrogen-fixing species isolated from cultivated soil. Int. J. Syst. Evol. Microbiol..

[58] Tikhonova E.N., Grouzdev D.S., Kravchenko I.K. (2019). *Azospirillum palustre* sp. nov., a methylotrophic nitrogen-fixing species isolated from raised bog. Int. J. Syst. Evol. Microbiol..

[59] Anandham R., Heo J., Krishnamoorthy R., SenthilKumar M., Gopal N.O., Kim S.J., Kwon S.W. (2019). *Azospirillum ramasamyi* sp. nov., a novel diazotrophic bacterium isolated from fermented bovine products. Int. J. Syst. Evol. Microbiol..

[60] Dos Santos Ferreira N., Hayashi Sant’ Anna F., Massena Reis V., Ambrosini A., Gazolla Volpiano C., Rothballer M., Schwab S., Baura V.A., Balsanelli E., Pedrosa F.O. (2020). Genome-based reclassification of *Azospirillum brasilense* Sp245 as the type strain of *Azospirillum baldaniorum* sp. nov. Int. J. Syst. Evol. Microbiol..

[61] Zhao Z.L., Ming H., Ding C.L., Ji W.L., Cheng L.J., Niu M.M., Zhang Y.M., Zhang L.Y., Meng X.L., Nie G.X. (2020). *Azospirillum thermophilum* sp. nov., isolated from a hot spring. Int. J. Syst. Evol. Microbiol..

[62] Hartmann A., Bashan Y. (2009). Ecology and application of *Azospirillum* and other plant growth-promoting bacteria (PGPB)—Special issue. Eur. J. Soil Biol..

[63] Martin-Didonet C.C.G., Chubatsu L.S., Souza E.M., Kleina M., Rego F.G., Rigo M.L.U., Yates M.G., Pedrosa F.O. (2000). Genome structure of the genus *Azospirillum*. J. Bacteriol..

[64] Wisniewski-Dyé F., Lozano L., Acosta-Cruz E., Borland S., Drogue B., Prigent-Combaret C., Rouy Z., Barbe V., Mendoza Herrera A., González V. (2012). Genome Sequence of *Azospirillum brasilense* CBG497 and Comparative Analyses of *Azospirillum* Core and Accessory Genomes provide Insight into Niche Adaptation. Genes.

[65] Kwak Y., Shin J.H. (2016). First *Azospirillum* genome from aquatic environments: Whole-genome sequence of *Azospirillum thiophilum* BV-S T, a novel diazotroph harboring a capacity of sulfur-chemolithotrophy from a sulfide spring. Mar. Genom..

[66] García J.E., Labarthe M., Pagnussat L., Amenta M., Creus C.M., Maroniche G.A. (2020). Signs of a phyllospheric lifestyle in the genome of the stress-tolerant strain *Azospirillum brasilense* Az19. Syst. Appl. Microbiol..

[67] Pérez Castañeda L.M., Cruz Hernández M.A., Mendoza Herrera A. (2011). Variabilidad genética de aislamientos no-típicos de *Azospirillum brasilense* por análisis PCR-RFLP del ADN 16S ribosomal. Phyton.

[68] Barkovskii A.L., Korshunova V.E., Pozdnyacova L.I. (1995). Catabolism of phenol and benzoate by *Azospirillum* strains. Appl. Soil Ecol..

[69] López-de-Victoria G., Lowell C.R. (1993). Chemotaxis of *Azospirillum* species to aromatic compounds. Appl. Environ. Microbiol..

[70] Eckford R., Cook D., Saul D., Aislabie J., Foght J. (2002). Free-Living Heterotrophic Nitrogen-Fixing Bacteria Isolated from Fuel-Contaminated Antarctic Soils. Appl. Environ. Microbiol..

[71] Nozawa M., Scow K.M., Rolston D.E. (2005). Reduction of Perchlorate and Nitrate by Microbial Communities in Vadose Soil. Appl. Environ. Microbiol..

[72] Muratova A.Y., Turkovskaya O.V., Antonyuk L.P., Makarov O.E., Pozdnyakova L.I.V., Ignatov V. (2005). Oil-Oxidizing Potential of Associative Rhizobacteria of the Genus *Azospirillum*. Microbiology.

[73] Miranda-Martínez M.R., Delgadillo-Martínez J., Alarcón A., Ferrera-Cerrato R. (2007). Degradación de fenantreno por microorganismos en la rizósfera del pasto alemán. Terra Latinoam..

[74] Cruz-Hernández M.A., Jimenez-Andrade J.M., Herrera A.M. (2019). Characterization of the degradation potential of xenobiotic compounds by the rhizobacteria *Azospirillum brasilense*. Mex. J. Biotechnol..

[75] Johnsen A.R., Wick L.Y., Harms H. (2005). Principles of microbial PAH-degradation in soil. Environ. Pollut..

[76] Chong H., Li Q. (2017). Degradación de fenantreno por microorganismos en la rizósfera del pasto alemán. Microb. Cell Fact..

[77] Nievas M.L., Commendatore M.G., Esteves J.L., Bucalá V. (2008). Biodegradation pattern of hydrocarbons from a fuel oil-type complex residue by an emulsifier-producing microbial consortium. J. Hazard. Mater..

[78] Hmidet N., Ayed H., Jacques P., Nasri M. (2017). Enhancement of Surfactin and Fengycin Production by *Bacillus mojavensis* A21: Application for Diesel Biodegradation. BioMed Res. Int..

[79] Supaphol S., Jenkins S.N., Intomo P., Waite I.S., O’Donnell A.G. (2011). Microbial community dynamics in mesophilic anaerobic co-digestion of mixed waste. Bioresour. Technol..

[80] Maimona S., Noshin I., Muhammad A., Muhammad S., Iftikhar A., Arghya B. (2021). Development of a plant microbiome bioremediation system for crude oil contamination. J. Environ. Chem. Eng..

[81] Xing S., Min D., Jiawei Y., Lin S., Xiao T., Changsheng P., Imran A. (2022). A critical review on the phytoremediation of heavy metals from environment: Performance and challenges. Chemosphere.

[82] Langenbach T., Nascimento A., Sarpa M. (1988). Influence of heavy metals on nitrogen fixation and growth of *Azospirillum* strains. Rev. Latinoam. Microbiol..

[83] Belimov A.A., Dietz K.J. (2000). Effect of associative bacteria on element composition of barley seedlings grown in solution culture at toxic cadmium concentrations. Microbiol. Res..

[84] Belimov A.A., Kunakova A.M., Safronova V.I., Stepanok V.V., Yudkin L.Y., Alekseev Y.V., Kozhemyakov A.P. (2004). Employment of rhizobacteria for the inoculation of barley plants cultivated in soil contaminated with lead and cadmium. Microbiology.

[85] Akond M.A., Khan Z.U. (2005). Role of carbon sources and heavy metals on growth and nitrogen fixing potential of *Azospirillum* and the effect of *Azospirillum strains* on vegetative growth of rice. J. Environ. Sci. Stud. (Dhaka).

[86] Kamnev Alexander A., Anna V.T., Lyudmila P.A., Petros A.T., Moschos G.P., Philip H.E. (2005). Effects of heavy metals on plant-associated rhizobacteria: Comparison of endophytic and non-endophytic strains of *Azospirillum brasilense*. J. Trace Elem. Med. Biol..

[87] Lyubun Y.V., Fritzsche A., Chernyshova M.P., Dudel E.G., Fedorov E.E. (2006). Arsenic transformation by *Azospirillum brasilense* Sp245 in association with wheat (*Triticum aestivum* L.) roots. Plant Soil.

[88] Vezza M.E., Olmos Nicotra M.F., Agostini E., Talano M.A. (2020). Biochemical and molecular characterization of arsenic response from *Azospirillum brasilense* Cd, a bacterial strain used as plant inoculant. Environ. Sci. Pollut. Res. Int..

[89] Ogar A., Sobczyk Ł., Turnau K. (2015). Effect of combined microbes on plant tolerance to Zn-Pb contaminations. Environ. Sci. Pollut. Res. Int..

[90] Ganesh K.S., Sundaramoorthy P., Nagarajan M. (2015). Organic soil amendments: Potential source for heavy metal accumulation. World Sci. News.

[91] Rojas Aparicio A., Vázquez J., Marbella J., Romero G.N., Rodríguez Barrera M.A., Toribio J.J., Romero R.Y. (2016). Evaluación de compost con presencia de metales pesados en el crecimiento de *Azospirillum brasilense* y *Glomus intraradices*. Rev. Mex. Cienc. Agric..

[92] Arora K., Sharma S., Monti A. (2016). Bio-remediation of Pb and Cd polluted soils by switchgrass: A case study in India. Int. J. Phytoremediation.

[93] Balakrishnan B., Sahu B.K., Kothilmozhian Ranishree J., Lourduraj A.V., Nithyanandam M., Packiriswamy N., Panchatcharam P. (2017). Assessment of heavy metal concentrations and associated resistant bacterial communities in bulk and rhizosphere soil of *Avicennia marina* of Pichavaram mangrove, India. Environ. Earth Sci..

[94] Xu Q., Pan W., Zhang R., Lu Q., Xue W., Wu C., Song B., Du S. (2018). Inoculation with *Bacillus subtilis* and *Azospirillum brasilense* Produces Abscisic Acid That Reduces Irt1-Mediated Cadmium Uptake of Roots. J. Agric. Food Chem..

[95] Armendariz A.L., Talano M.A., Olmos N.M.F., Escudero L., Breser M.L., Porporatto C., Agostini E. (2019). Impact of double inoculation with *Bradyrhizobium japonicum* E109 and *Azospirillum brasilense* Az39 on soybean plants grown under arsenic stress. Plant Physiol. Biochem..

[96] Pan W., Lu Q., Xu Q.R., Zhang R.R., Li H.Y., Yang Y.H., Liu H.J., Du S.T. (2019). Abscisic acid-generating bacteria can reduce Cd concentration in pakchoi grown in Cd-contaminated soil. Ecotoxicol. Environ. Saf..

[97] Vázquez A., Zawoznik M., Benavides M.P., Groppa M.D. (2021). *Azospirillum brasilense* Az39 restricts cadmium entrance into wheat plants and mitigates cadmium stress. Plant Sci..

